# Insights From Early Clinical Trials Assessing Response to mRNA SARS-CoV-2 Vaccination in Immunocompromised Patients

**DOI:** 10.3389/fimmu.2022.827242

**Published:** 2022-03-04

**Authors:** Frédéric Baron, Lorenzo Canti, Kevin K. Ariën, Delphine Kemlin, Isabelle Desombere, Margaux Gerbaux, Pieter Pannus, Yves Beguin, Arnaud Marchant, Stéphanie Humblet-Baron

**Affiliations:** ^1^Laboratory of Hematology, GIGA-I3, University of Liege and Centre Hospitalier Universitaire (CHU) of Liège, Liege, Belgium; ^2^Department of Medicine, Division of Hematology, Centre Hospitalier Universitaire (CHU) of Liège, Liège, Belgium; ^3^Virology Unit, Institute of Tropical Medicine Antwerp, Antwerp, Belgium; ^4^Department of Biomedical Sciences, University of Antwerp, Antwerp, Belgium; ^5^Department of Nephrology, Dialysis and Renal Transplantation, Hôpital Erasme, Université libre de Bruxelles, Brussels, Belgium; ^6^Scientific Directorate Infectious Diseases in Humans, Sciensano, Brussels, Belgium; ^7^Institute for Medical Immunology and ULB Center for Research in Immunology (U-CRI), Université libre de Bruxelles (ULB), Gosselies, Belgium; ^8^Department of Microbiology, Immunology and Transplantation, Laboratory of Adaptive Immunology, KU Leuven, Leuven, Belgium

**Keywords:** vaccine, BNT162b2, MRNA-1273, SARS-CoV-2, COVID-19, immunosuppressed, transplantation, inborn errors of immunity

## Abstract

It is critical to protect immunocompromised patients against COVID-19 with effective SARS-CoV-2 vaccination as they have an increased risk of developing severe disease. This is challenging, however, since effective mRNA vaccination requires the successful cooperation of several components of the innate and adaptive immune systems, both of which can be severely affected/deficient in immunocompromised people. In this article, we first review current knowledge on the immunobiology of SARS-COV-2 mRNA vaccination in animal models and in healthy humans. Next, we summarize data from early trials of SARS-COV-2 mRNA vaccination in patients with secondary or primary immunodeficiency. These early clinical trials identified common predictors of lower response to the vaccine such as anti-CD19, anti-CD20 or anti-CD38 therapies, low (naive) CD4^+^ T-cell counts, genetic or therapeutic Bruton tyrosine kinase deficiency, treatment with antimetabolites, CTLA4 agonists or JAK inhibitors, and vaccination with BNT162b2 versus mRNA1273 vaccine. Finally, we review the first data on third dose mRNA vaccine administration in immunocompromised patients and discuss recent strategies of temporarily holding/pausing immunosuppressive medication during vaccination.

## Introduction

Vaccination remains one of the most efficient ways to prevent infections (1). It is generally accepted that vaccines act by inducing pathogen-specific immune responses leading to the generation of protective antibodies (Ab) and of memory immune cells (2). In the last decades, correlates of protection induced by vaccines have been extensively studied (3). It has been postulated that the presence of pathogen-specific Ab and long-lived plasma and effector memory B cells are required to neutralize toxins or protect from infection caused by pathogens with a short incubation period. In contrast, the presence of central memory B and/or T cells might be sufficient to prevent infections caused by pathogens with a longer incubation period (3). Correlates of protection against SARS-CoV-2 have been elegantly studied in non-human primates. McMahan et al. demonstrated that SARS-CoV-2 naïve rhesus macaques could be protected from SARS-CoV-2 challenge by adoptive transfer of purified IgG from convalescent macaques (4). This protection was dose dependent. Interestingly, in situations of sub-protective Ab titers, depletion of CD8^+^ T cells in convalescent animals favored SARS-CoV-2 infection indicating the role of cell-mediated immunity when protecting Ab titers are suboptimal. More recently, a study of SARS-CoV-2 infection in nonhuman primates immunized with various doses of mRNA-1273 vaccine (vide infra) demonstrated a 10-fold reduction in viral replication in bronchoalveolar lavage and nasal swabs with 10-fold increase in the spike-specific binding titers (5). Interestingly, protection was achieved with significantly lower titers in the lower than in the upper respiratory tract. In humans, several studies support the hypothesis that serum titers of binding and neutralizing Ab are associated with protection as observed in nonhuman primates (6–9). In a recent large study of patients given the mRNA-1273 vaccine, 50% inhibitory dilution (ID50) neutralizing Ab titers on day 57 after vaccination correlated with vaccine efficacy defined as absence of vaccine breakthrough COVID-19 in the following 3 months (9). Specifically, in patients with undetectable ID50 at day 57, vaccine efficacy was 51%. In contrast, vaccine efficacy was 78%, 91% and 96%, respectively, in patients with day 57 value of 10, 100 and 1000 IU_50_/mL, respectively. Similarly, day 57 spike binding IgG of 33, 300 and 4000 binding antibody units (BAU)/mL were associated with 85%, 90% and 94% protection, respectively (it should be noted that these cut-of values of binding Ab differ most probably for delta and even more for omicron variant). Unfortunately, cellular correlates of protection were not assessed in that study.

General mechanisms of immunogenicity of T-cell dependent vaccines have been partly elucidated in the last decades. Briefly, following primary immunization, naive CD4^+^ T cells in the draining lymph node recognize a peptide presented at the surface of antigen presenting cells (APC, generally dendritic cells) in their major histocompatibility complex (MHC)-class II complex by the engagement of their T-cell receptor. A second signal produced by the interaction between CD80 and CD86 at the surface of the APC and CD28 on T cells allows the differentiation of pre-follicular helper T (T_FH_) cells followed by the formation of a germinal center reaction (10). Within germinal centers, antigen-activated B cells undergo hypersomatic mutations of immunoglobulin genes leading to the generation of high-affinity B cell clones that are positively selected and eventually differentiate into long-lived plasma cells and effector memory B cells. T_FH_ cells play a critical role in germinal center reactions by providing IL-21 and CD40 (and at later stage IL-4) signals to activated B cells assuring their survival, proliferation and differentiation (11). T_FH_ differentiation requires IL-6 and ICOS-L and is inhibited by IL-2. While dendritic cells provide the required signals for early T_FH_ differentiation, activated B cells are the critical APC for further T_FH_ differentiation as well as for T_FH_ expansion. Thus, efficient germinal center reaction involves a positive feedback loop between T_FH_ and high-affinity antigen-specific B cells. High affinity antigen-specific B cells lead to potent neutralizing Ab. Further, APC in the draining lymph node also induce a CD8^+^ T-cell response *via* presentation of pathogen antigens in their MHC-class I complex. Pathogen-specific CD4^+^ T cells further stimulate the CD8^+^ T-cell response.

Virtually all steps of the vaccine response to a neo-antigen can be the target of immunosuppressive or chemotherapeutic agents and/or can be impaired by cellular defects caused by primary or secondary immunodeficiency (12). For example, defective naive CD4^+^ T cells are observed in several conditions such as following allogeneic hematopoietic stem cell transplantation (13, 14) (allo-HCT), in people living with the HIV (PLWH) (15), or in elderly frail patients (16). TCR signaling in naive CD4^+^ T cells is impacted by calcineurin and mTOR inhibitors (17, 18). Efficient activation of naive CD4^+^ T cells by APC can be abrogated by CTLA4 analogs such as Abatacept or Belatacept, and to a lesser extent by mTor inhibitors [such as rapamycin or azithromycin that decrease CD28 expression by naive CD4^+^ T cells (19, 20)] and immunosenescence which is characterized by a reduced expression of CD28 by T cells (21). A large but incomplete (i.e. persistence of some memory B cells) deficit in B cell populations lasting up to 9-12 months has been observed following administration of anti-CD20 monoclonal antibodies (referred later as anti-CD20 therapy) such as rituximab (22, 23). Further, since B-cell pool recovery after anti-CD20 therapy follows their lineage ontogeny, it initially involves the expansion of functionally immature B cells (22). B cell functions are also inhibited by Bruton’s tyrosine kinase inhibitors, by CD19-targeting chimeric antigen receptor (CAR) T cells, and are obviously absent in patients with x-linked agammaglobulinemia (XLA). In addition, B and T cell proliferation, a critical component of vaccine responses, is affected by several chemotherapeutic/immunosuppressive medications such as antimetabolites (fludarabine, methotrexate or mycophenolate mofetil (MMF)) which inhibit DNA synthesis preferentially in lymphocytes. Finally, Janus kinase (JAK) inhibitors inhibit the signaling of key cytokines involved in vaccine responses such as IL-2 and IL-21.

## Immune Response to mRNA Vaccination in Animal Models

The immunogenicity of nucleoside-modified mRNA (i.e. by eliminating pathogen-associated molecular patterns *via* incorporation of 1-methylpseudouridine, m1Ψ) encapsulated in lipid nanoparticles (mRNA-LNPs) vaccines has been studied in animal models by Pardi et al. (24–26). In mice, following intradermal vaccination, m1Ψ-mRNA-LNPs vaccines are incorporated in skin resident APC through endocytosis. APC then migrate into the draining lymph nodes. Intracellular mRNA translation in the APC results in a strong protein production that gradually decreases over a 13-day period (of note, nucleoside modification is required for sustained and high protein production). Following antigen protein synthesis, antigenic proteins are degraded in small peptides by the proteasomes and are then presented by the MHC class I and class II to cytotoxic CD8^+^ and helper CD4^+^ T cells, respectively (27). This results in the proliferation of polyclonal antigen-specific CD4^+^ T cells differentiating in T_FH_ cells stimulating germinal center B cells producing neutralizing Ab, and in the differentiation of T helper (T_H_)1 CD4^+^ cells. On the other hand, antigenic peptides presented by APC through MHC class I molecules stimulate an antigen-specific CD8^+^ T cell response that is further enhanced by CD4^+^ helper T cells. It should be emphasized that LNPs, in addition to their role of carrier, act as a strong adjuvant (26). Although it remains to be elucidated which innate immune signals are induced by LNP adjuvant, a recent study demonstrated upregulation of interferon signaling and high levels of inflammatory cytokines IL-6, IL-1β and granulocyte-macrophage colony-stimulating factor (GM-CSF) following LNP injection in mice, together with massive neutrophil infiltration (28).

A recent study compared vaccination with SARS-CoV-2 mRNA-LNP versus SARS-CoV-2 receptor-binding domain (RBD) protein plus MF-59-like adjuvant (a “classical” vaccination platform) (29). The authors demonstrated that SARS-CoV-2 mRNA-LNP vaccination led to a high T_FH_ response and potent germinal center reactions leading to a high production of neutralizing Ab. In contrast, only weak germinal center reactions were induced by the adjuvanted protein vaccine. Importantly, SARS-CoV-2 mRNA-LNP (but not protein-adjuvanted) vaccination induced high amounts of long-lived plasma cells and memory B cells.

Injection of m1Ψ-mRNA-LNPs vaccine in rhesus macaques also induced a strong T_FH_ response in the draining lymph node seven days after vaccination, indicating that the immunogenicity of the vaccine is consistent in rodents and in non-human primates (24). The potent T_FH_ response in macaques resulted in the production of high titers of neutralizing Ab that persisted over a 12-month period after vaccination.

### Immunogenicity of the Anti SARS-Cov-2 BNT162b1, BNT162b2, and mRNA-1273 Vaccines in Animal Models

The immunogenicity of two m1Ψ-mRNA-LNPs vaccines against SARS-CoV-2 developed by BioNTech/Pfizer was first assessed in preclinical models (30). Modified mRNA in BNT162b1 vaccine encodes a soluble secreted trimerized receptor-binding domain (RBD) while the modified mRNA in BNT162b2 vaccine encodes the full-length trans-membrane S glycoprotein that is stabilized in the prefusion conformation. Indeed, prior works showed that a prefusion-stabilized MERS-CoV protein was more immunogenic than the wild-type MERS-CoV secreted spike protein (31). In a mouse model of a single intramuscular injection, both vaccines induced high titers of binding and of neutralizing Ab together with strong T_H_1 polarized CD4^+^ T cell, T_FH_ cell and CD8^+^ T cell responses. These responses were dose-dependent. CD8^+^ T cell responses were characterized by CD8^+^ T cells producing IL-2 and/or interferon gamma (IFNγ) and were somewhat higher following administration of the BNT162b2 vaccine. Comparable observations were made in a macaque model in which both vaccines induced high amounts of neutralizing Ab, a T_H_1-biased response with high frequencies of vaccine-specific CD4^+^ T cells producing IFNγ, IL-2 or TNF and low frequencies of CD4^+^ T cells producing the T_H_2 cytokines, and high frequencies of CD8^+^ T cells producing IFNγ. Finally, the BNT162b2 vaccine provided a slightly better protection against SARS-CoV-2 than the BNT162b1 vaccine.

mRNA-1273 is an mRNA-LNPs vaccine encoding prefusion-stabilized SARS-CoV-2 spike (2P) protein (32). Two intramuscular injections of mRNA-1273 vaccine at a 3-week (mouse models) or 4-week (non-human primate models) interval induced high levels of neutralizing Ab (32–34). As observed with the BNT162b vaccines, Ab response following mRNA-1273 vaccination was dose-dependent. In the primate model, animals given the 100-μg dose developed a T_H_1 cell response four weeks after the second injection. In contrast, T_H_2 response was either not observed or weak. This is of importance given that vaccine-enhanced respiratory disease has been associated with a T_H_2 biased immune response (34). There was also indirect evidence of a T_FH_ reaction since IL-21 [the canonical cytokine secreted by T_FH_ (35, 36)] was elevated four weeks after the second injection in a dose-dependent fashion (33). Interestingly, a strong virus-specific CD8^+^ T cell response was observed following mRNA-1273 vaccination in mice but not in the non-human primate model (32, 33). In addition, rhesus macaques immunized with two injections of 100 μg mRNA-1273 experienced antibody waning mainly the first six months after immunization (37). Specifically, specific binding Ab decreased from ∼3000 BAU/mL at week 6 to ∼260 BAU/mL at week 24 and ∼188 BAU/mL at week 48. Following SARS-CoV-2 B.1.351 Beta variant challenge at 48 weeks after first immunization, four days were required to achieve undetectable virus in the broncho-alveolar lavage (versus two days when the challenge was performed early after vaccination). The clearance of virus in the lung was concomitant to the occurrence of an anamnestic Ab response in the broncho-alveolar lavage. Remarkably, booster mRNA-1273 vaccination at six months following vaccination restored peak frequencies of memory B cells and induced a 20-fold increase in neutralizing Ab against wild-type and Beta SARS-CoV-2 variant (38). The booster vaccination also induced spike-specific T_H_1 (but not T_H_2) and T_FH_ responses. Interestingly, neutralizing Ab titers against SARS-CoV-2 Beta variant, Ab repertoire and SARS-CoV-2 β variant replication in primates were almost identical after boosting with the homologous mRNA-1273 vaccine or an heterologous mRNA-1273.β vaccine which encodes the spike sequence of the Beta variant.

## Immune Response to SARS-COV-2 MRNA Vaccination in Healthy Adults

### B-Cell and T-Cell Immunity

As observed in animal models, two injections of the BNT162b2 vaccine in healthy humans induced strong Ab responses with anti-RBD Ab titers 3.5 times higher than what was observed in patients who had recovered from COVID-19 (39). The Ab response after vaccination was higher in recovered COVID-19 patients who did not seem to benefit from the second vaccine administration (40). However, a recent study revealed that most vaccine-induced Ab did not have neutralizing activity and that the ratio of binding to neutralizing Ab was higher following mRNA vaccination than following natural infection (41). Anti-RBD Ab and neutralizing Ab titers decreased progressively over time but remained detectable in the vast majority of subjects beyond six months after vaccination (42). The vaccine also induced strong RBD-specific T_H_1 (expressing IL-2 and/or IFNγ but not IL-4) and T_FH_ responses, as well as expansion of RBD-specific CD8^+^ T cells expressing IL-2 and/or IFNγ (39, 43). Interestingly, a sustained (i.e. persisting for at least six months) specific T_FH_ response was also demonstrated in the draining lymph node of the injection site (44). Importantly, the T cell response was polyclonal and targeted several epitopes that were conserved most frequently in SARS-CoV-2 variants (43, 45). While all patients mounted CD4^+^ T cell specific responses, a specific CD8^+^ T cell response was only observed in 71% and 88% naïve subjects following one and two vaccine injections, respectively (46). Comparable observations were made following two injections of 25 μg of mRNA-1273 vaccine (47). Numbers of antigen-specific CD4^+^ and CD8^+^ T cells contracted from peak response to three months post vaccination. Then, while specific CD4^+^ T cells remained stable, specific CD8^+^ T cells continued to decline in the subsequent three months. Interestingly, there was a strong correlation between antigen-specific T_H_1 cell frequencies after the first injection and the frequencies of antigen-specific CD8^+^ T cells after the second injection (46). Similarly, there was a strong correlation between antigen-specific circulating T_FH_ cell frequencies after the first injection and neutralizing Ab titers after the second injection (42, 46).

Generation of memory B cells is probably critical for long-term protection against SARS-CoV-2. Interestingly, following natural SARS-CoV-2 infection, RBD-specific memory B cells persist for at least six months and continue to evolve acquiring higher somatic hypermutation and higher potency, possibly because of antigen persistence (48, 49). As observed with natural SARS-CoV-2 infection, two injections of the BNT162b2 or mRNA-1273 vaccine induced RBD-specific memory B cells including memory B cells specific for the Alpha, Beta and Delta variants (42). As seen for Ab response, recovered COVID-19 patients had markedly higher numbers of RBD-specific memory B cells than naïve subjects following vaccination at early time points (50). However, while RBD-specific memory B cell frequency increased from three to six months after vaccination in naïve subjects, they declined over time in convalescents.

Several reports have suggested higher immunogenicity of mRNA-1273 than BNT162b vaccine [and lower immunogenicity of Ad26.COV2.S than BNT162b vaccine (51)] among healthy subjects (52–54). A possible explanation is the higher dose of mRNA in the mRNA-1273 (100 μg) than in the BNT162b (30 μg) vaccine. In addition, a recent study still in pre-print format showed higher epitope spreading, IgA immunity and specific Ab function (*i.e.* enhanced Ab-dependent neutrophil phagocytosis and Ab-dependent NK cell degranulation) in patients vaccinated with the mRNA-1273 in comparison to the BNT162b vaccine (55). In concordance with the higher immunogenicity of mRNA-1273 vaccine, a recent study observed lower 24-week COVID-19 outcomes (defined as documented SARS-CoV-2 infection, symptomatic COVID-19, hospitalization and/or admission for ICU for COVID-19, or death from COVID-19) in persons vaccinated with the mRNA-1273 vaccine than in those given the BNT162b2 vaccine (56).

In summary, current data indicate that, in SARS-CoV-2 naïve healthy subjects, first injection of the mRNA vaccine activates specific T_H_1 CD4^+^ T cells and specific T_FH_ cells. T_H_1 T cells may promote the generation of specific CD8^+^ T cells while specific T_FH_ cells promote germinal center responses, the production of neutralizing Ab and the differentiation of memory B cells. While Ab titers and T-cell frequencies decrease over time after vaccination, specific memory B cells continue to mature and increase in number during the first six months after vaccination. In addition, while both mRNA-1273 and BNT162b vaccines induce comparable immune reactions, higher binding and neutralizing Ab titers are observed with mRNA-1273 than with BNT162b vaccines.

### Systems Vaccinology Analysis of SARS-CoV-2 mRNA Vaccine Responses

System biology approaches have paved the way to provide a global picture of immune responses to vaccines (57–59). A systems SARS-CoV-2 mRNA vaccinology study performed in 56 healthy subjects has been reported recently (60). This study confirmed the rapid induction of neutralizing Ab following injection of the BNT162b2 vaccine and that this Ab response was boosted by the second injection. Furthermore, as observed previously by other groups of investigators, analyses performed seven days after second vaccination demonstrated the generation of spike-specific T_H_1 and to a much lower extent T_H_2 cells as well as spike specific CD8^+^ T cells mostly expressing IFNγ and TNF.

While gene-set enrichment analysis (GSEA) of bulk RNAseq analyses demonstrated that both vaccine doses induced interferon and antiviral response modules, the second dose of the vaccine induced a much higher immune response compared to the first one with upregulation of dendritic cell activation, Toll-like receptor signaling, and monocyte and neutrophil modules in GSEA analyses. In addition, flow cytometry analyses on whole blood demonstrated increasing frequencies of inflammatory (CD14^+^CD16^+^) monocytes two days after each vaccine and higher levels of phosphorylated STAT1 (pSTAT1) and STAT3 (pSTAT3) in various immune cell types the day after the second vaccination as compared with the day after the first vaccination. Finally, IFNγ and CXCL10 serum concentration were increased as compared to baseline on days 1 and 2 after first vaccination, but were further increased after the booster dose. A recent study supports these observations and identified a systemic IL-15, IFNγ, and IP-10/CXCL10 signature associated with BNT162b2 vaccine response (61).

Taken together, these findings underline the role of innate immunity in the response to SARS-CoV-2 mRNA vaccine and suggest that priming of the innate immune system allows for a higher immune response after the second immunization. In line with this hypothesis, recent studies observed higher (62) [or a trend for higher (63)] binding Ab following BNT162b2 vaccination in patients with multiple sclerosis treated with INF-beta 1a. It should be noted that activation of the innate immune system also occurs following natural infection with SARS-CoV-2, and that aberrant monocyte and neutrophil activation has been associated with COVID-19 severity (64).

## Immune Response to SARS-COV-2 MRNA Vaccination in Immunocompromised Patients

A number of studies have confirmed that immunocompromised patients are at high risk of developing severe COVID-19 disease and of dying when infected with SARS-CoV-2 (65–68). This seems to be particularly the case in patients with combined defects of B and T cell immunity as observed early after transplantation as well as in patients given intensive chemotherapy (69–73). Anti-CD20 therapy inducing a profound B-cell depletion also increases the risk of severe COVID-19 and death (74–76). Interestingly, in patients on anti-CD20 therapy for an hematological malignancy, those able to mount an effective CD8^+^ T cell response to the virus are less likely to succumb to SARS-CoV-2 infection (77). This suggests that SARS-CoV-2-specific T-cell responses are able to clear the virus in patients with limited/absent B-cell immunity. Among people living with HIV, low CD4^+^ T cell counts were associated with higher COVID-19 severity (78). Finally, both allo-HCT recipients and patients given anti-CD20 therapy are at risk of longer duration of shedding and of COVID-19 relapses, often associated with virus replication and mutation (79).

Although early administration of COVID-monoclonal Ab most probably improves the outcome of immunosuppressed patients infected by SARS-CoV-2 when administered early in the disease course (80, 81), it remains critical to protect these patients with effective vaccination. This is challenging since, as described above, response to mRNA vaccination requires the cooperation of many components of the immune system. In the following paragraphs we will review available data on SARS-CoV-2 mRNA vaccination in immunocompromised patients, with the aim of identifying common risk factors for poor/no response to the vaccine.

### People Living With HIV (PLWH)

First reports showed that most PLWH responded to mRNA vaccination in terms of binding and neutralizing Ab (82). This was consistent with observations made in a simian animal model (83). However, two recent reports stressed that patients with advanced HIV infection had lower response to mRNA vaccination (15, 84). Hassold et al. retrospectively compared binding Ab response to SARS-CoV-2 vaccination in PLWH with CD4^+^ T cell counts < 500/μL versus in those with CD4^+^ T cell counts ≥ 500/μL (15). Median patient age was 54 years. Vaccines included BNT162b2 (75%), mRNA-1273 (8.5%) and ChAdOx1 nCoV-19 (16.5%). Six patients with CD4^+^ T cell counts < 400 cells/μL failed to seroconvert including 4 of 18 patients with CD4^+^ T cell counts < 200 cells/μL while all patients with CD4^+^ T cell counts > 400 cells/μL seroconverted. Median binding Ab levels were 624 BAU/mL, 397 BAU/mL (P=0.046), and 248 BAU/mL (P=0.002) in patients with CD4^+^ T cell counts > 500 cells/μL (n=51), between 200 and 500 cells/μL (n=36), and < 200 cells/μL (n=18), respectively.

Spinelli et al. investigated predictors of binding Ab response in 100 PLWH and 100 controls given either the mRNA1273 (25%) of the BNT162.b2 (75%) vaccine (84). Median age was 59 years in both cohorts. Twelve PLWH failed to achieve anti-spike binding IgG. They were all given the BNT162b2 vaccine (P=0.03). These included 7 out of 7 patients with CD4^+^ T cell counts < 200 cells/μL. Further, there were 28% higher anti-spike IgG levels for each 100-cell increase in CD4^+^ T-cell count.

Taken together, these two studies stress the pivotal role of CD4^+^ T cells for Ab response to mRNA vaccines.

### Patients With Chronic Inflammatory Disease

The treatment of chronic inflammatory diseases (CID) such as multiple sclerosis, inflammatory bowel disease, systemic lupus erythematous, psoriasis, rheumatoid arthritis or systemic sclerosis often involves immunosuppressive medications. The impact of these medications on Ab response to SARS-CoV-2 mRNA vaccination has been recently assessed in large studies (85–89). Specifically, Furer et al. assessed the response to two doses of the BNT162b2 vaccine in a cohort of 686 patients with autoimmune inflammatory rheumatic disease (AIIRD) versus 121 healthy controls (85). Median patient age was 59 years. Seroconversion rate was 86% in AIIRD patients and 100% in healthy controls. In multivariate analyses, lower immunogenicity was observed in patients > 65 years old (OR=0.43, P=0.002) as well as in those on glucocorticoids (OR=0.48, P=0.02), anti-CD20 therapy (OR=0.13, P<0.001), mycophenolate mofetil (MMF; OR=0.1, P=0.0013)), and those on the CTLA-4 agonist abatacept (HR=0.14, P<0.001). Deepak et al. reported detectable anti-spike Ab responses in 89% (118/133) of patients with CID (mean age 45.5 years) and in 100% (53/53) of healthy controls following the administration of two doses of SARS-CoV-2 mRNA vaccine (no data were available on the proportion of patients given the BNT162b2 or the mRNA1273 vaccine). Among CID patients, those on glucocorticoids therapy [seroconversion in 11/17 (65%)] and those given anti-CD20 therapy [seroconversion in 6/10 (60%)] had significantly lower Ab titers. No statistically significant associations were observed for other immunosuppressive medications such as antimetabolites or JAK inhibitors. A meta-analysis including data from 25 studies of SARS-CoV-2 mRNA vaccination in CID patients assessed the impact of treatment on seroconversion rates (89). The study identified that seroconversion after two doses of the mRNA vaccine was > 90% in patients with anti-TNF, anti-IL-17 or anti IL-6 therapy, between 70 and 90% in those on corticosteroids, JAK inhibitors or the anti-metabolite MMF, and < 70% in those given anti-CD20 therapy and those on CTLA-4 agonist therapy (abatacept). Unfortunately, the study did not compare seroconversion rates nor Ab titers according to the type of vaccine received. Despite these encouraging seroconversion rates, it should be noted that patients on anti-TNF therapy have lower neutralizing Ab levels (especially against the B.1.617.2 Delta variant) than healthy subjects (90). Finally, a recent analysis assessed the development of neutralizing Ab and T cell response to BNT162b2 vaccine in 64 patients with CID (median age 52 years) (91). Neutralizing Ab against the Alpha and the Delta variants were observed in 100% and 100% of the controls, respectively, 5% and 0% of patients on anti-CD20 therapy, respectively, and 87% and 57% of those on methotrexate, respectively. In addition, patients on methotrexate had lower T cell response to the vaccine.

The impact of anti-CD20 therapy on the response to SARS-CoV-2 mRNA vaccination has been studied in detail (92, 93). A large study compared responses to SARS-CoV-2 mRNA vaccine in 29 healthy controls versus 96 patients with a history of anti-CD20 therapy (mainly rituximab) given as treatment of autoimmune disease (n=71), malignancy (n=6) or for induction of ABO-incompatible kidney transplantation (n=19) (92). Anti-CD20 therapy was given for a median of 1 year (IQR 0.5-2.6 years) before vaccination and most patients had other immunosuppressive medication at the time of the vaccination. The authors observed that time since last anti-CD20 therapy (> 7.6 months), as well as higher B and CD4^+^ T cell counts were associated with higher Ab response. More recently, an elegant study compared humoral and cellular responses to SARS-CoV-2 mRNA vaccination in 10 controls and 20 multiple sclerosis patients treated with anti-CD20 therapy [ocrelizumab (n=19) or rituximab (n=1)] (93). The authors observed that anti-CD20 therapy preserved the antigen-specific T_H_1 response but reduced T_FH_ cell responses as well as anti-RBD Ab and RBD-specific memory B cell responses. Intriguingly, antigen-specific CD8^+^ T cell responses were increased in patients on anti-CD20 therapy. An important unsolved question is whether efficient CD8^+^ T cell responses to vaccination provide sufficient protection in patients with limited humoral responses to the vaccine.

### Patients With Hematological Malignancy

**Figure 1:**
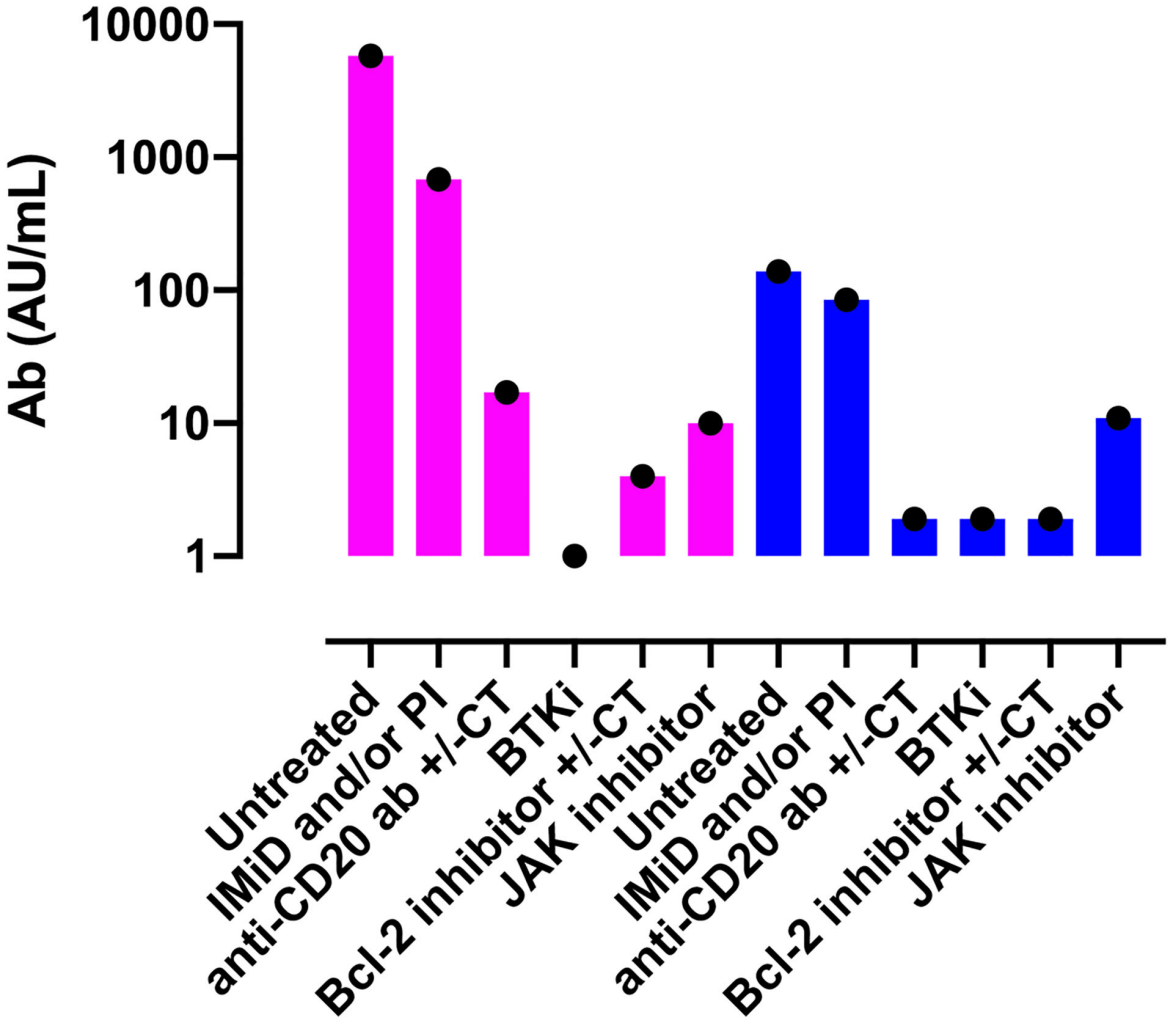
Serological responses to two doses of the BNT162b2 vaccine according to current treatment in patients with hematological malignancies. Violet bars show data from the study by Maneikis et al. (94) in which the Abott Architect SARS-CoV-2 IgG Quant II immunoassay was used to detect anti-RBD Ab (samples with < 50 AU/mL were considered seronegative) while blue bars show the data from the study from Tzarfati et al. (95) in which SARS-CoV-2 S1/S2 IgG test (samples with < 12 AU/mL were considered seronegative) was used to assess humoral response. Please note that the titers from the two studies cannot be compared given that different serological tests were used. Ab, antibodies; IMiD, Immunomodulatory imide drug; PI, proteasome inhibitor; anti-CD20 Ab, monoclonal antibody targeting CD20 (such as rituximab); BTKi, Bruton tyrosine kinase inhibitor; CT, chemotherapy.

Maneikis et al. reported Ab responses following two doses of BNT162b2 vaccine in a large cohort of patients suffering from a hematological malignancy (857 patients anti-S1 IgG negative at first vaccination) as well as in 67 sero-negative healthy controls (94). The authors observed that in comparison to patients with untreated hematological malignancy (n=53, median 5761 AU/mL) or healthy controls (n=67, median 6961 AU/mL), significantly lower anti-S1 IgG Ab were observed after two doses of the BNT162b2 vaccine in patients given B-cell targeting therapy such as anti-CD20 Ab (n=87, median 17 AU/mL), Bruton’s tyrosine kinase inhibitor (n=44, median 0 AU/mL), or the Bcl-2 inhibitor venetoclax (n=10, median 4 AU/mL). Similarly, patients on JAK-1/2 inhibitor (n=16, median 10 AU/mL) and those with immunomodulatory imide drugs with or without proteasome inhibitor (n=76, median 679 AU/mL) also had lower binding Ab titers (**Figure 1**). Comparable results were reported by Tzarfati et al. in a cohort of 423 patients suffering from a hematological malignancy (95). The authors observed that the seroconversion rate after two vaccine doses was 86% in untreated patients, 0% in those given single agent anti-CD20 therapy, 25% in patients on Bcl-2 inhibitors, 40% in patients on Bruton’s tyrosine kinase inhibitors, and 42% in those on JAK1/2 inhibitors (**Figure 1**). In addition, another large study demonstrated a strong correlation between binding anti-S IgG levels on day 42 and B-cell counts at baseline (Spearman r=0.677, P<0.0001), but not with baseline CD4^+^ or CD8^+^ T cell counts (96). Importantly, it should be noted that in patients with B-cell malignancies, approximately 75% of patients who fail to seroconvert after two doses of mRNA vaccine have a detectable T-cell response against the spike protein (97).

In addition to these large studies involving patients with diverse hematological malignancies, several studies assessed the humoral response to anti-SARS-CoV-2 mRNA vaccine in patients with a specific malignancy. The most relevant ones for this review are summarized in the following paragraphs.

#### Chronic Lymphocytic Leukemia

A large multicenter study assessed response to BNT162b2 vaccine in a cohort of 167 patients with chronic lymphocytic leukemia (CLL) (98). Median patient age was 71 years. While all 52 healthy controls achieved a humoral response following two doses of the vaccine, this was the case in only 39.5% of CLL patients. Seroconversion rate was 55% in treatment naïve patients (demonstrating that the disease itself impaired immune response to mRNA vaccines) and only 16% in those under treatment at the time of vaccination. Further, none of the patients given anti-CD20 therapy within 12 months before vaccination had a detectable humoral response (98). In multivariate analysis, seroconversion was more frequently observed in patients ≤ 65 years old, in females, in patients not on active therapy and in patients with IgG levels > 550 mg/dL. Parry et al. observed a markedly negative impact of receiving Bruton tyrosine kinase inhibitor or anti-CD20 therapy within the year before vaccination on serological response rates to SARS-CoV-2 mRNA (BNT162b2, n=204) or adenoviral (ChAdOx1 nCov-19, n=296) vaccine in a large cohort of 500 patients (median age 67 years) with CLL in which 67% of the patients acquired anti-spike IgG (versus 100% in healthy controls) (99). Importantly, CLL patients with a serological response had markedly lower neutralizing Ab against the Delta SARS-CoV-2 variant than healthy controls. Bagacean et al. assessed the response to mRNA vaccine (71% BNT162b2, 14% mRNA-1273, 15% non-specified mRNA) in 530 CLL patients. Median patient age was 71 (100). Fifty-two % of the patients achieved anti-spike binding IgG seroconversion. Seventy-two % of treatment-naive patients responded versus 52% of those given venetoclax monotherapy, 22% in those given Bruton tyrosine kinase inhibitor monotherapy, and 0% in those given venetoclax plus anti-CD20 therapy. In multivariate analysis, age > 65 years (P=0.02), ongoing CLL treatment (P<0.001) and gamma globulins ≤ 6 g/L (P=0.03) were associated with lower serological response.

#### Non-Hodgkin Lymphoma

Perry et al. assessed the humoral response to two doses of BNT162b2 vaccine in patients with B-cell non-Hodgkin lymphoma (n=149, median age 64 years). While 98.5% of healthy controls achieved seroconversion, a serologic response was detected in 89% of treatment naïve patients, in 7% of those actively treated with anti-CD20 therapy, and 67% in those treated by anti-CD20 therapy > 6 months before vaccination (101). Comparable results were reported by Ghione et al. who observed that only 6 out of 52 patients given anti-CD20 therapy < 9 months before vaccination responded while most patients vaccinated > 9 months after anti-CD20 therapy responded to anti-SARS-CoV-2 vaccines (BNT162b2 (n=40) or mRNA1273 (n=45) (102).

#### Multiple Myeloma

A first study reported that among patients with multiple myeloma, 7 out of 14 (50%) patients on anti-CD38 therapy seroconverted after two doses of the BNT162b2 vaccine versus 26 out of 28 (93%) patients on anti-myeloma treatment without anti-CD38 therapy (i.e. proteasome inhibitor +/- Imids, P=0.003) (103). A subsequent large study (n=320) reported a response rate of 84% after two doses of mRNA vaccine (69% BNT162.b2, 27% mRNA-1273, 4% unknown) in multiple myeloma patients (median age 68 years) (104). However, Ab titers were highly variable. Factors associated with absence of serological response in multivariate analyses included anti-CD38 (OR=4.3, P=0.005) and anti-BCMA (OR=10.3, P<0.001) therapies. It should be noted that asymptomatic and untreated patients with smoldering myeloma have also a lower response to BNT162b2 vaccine than healthy controls and that there is a correlation between more advanced smoldering myeloma and lower humoral response to SARS-CoV-2 vaccination (105).

### Allogeneic Stem Cell Transplant and CART Cell Recipients

It is now well established that successful immune recovery takes several months to years following allo-HCT (106, 107). Indeed, most circulating T cells during the first months after transplantation arise through peripheral homeostatic expansion of T cells infused with the graft. Beyond day 100, neo-generation of T cells by the thymus progressively plays an increasing role in reconstituting the T-cell pool, but only in patients < 60 years of age at transplantation (13, 14, 108, 109). Consequently, CD4^+^ T-cell counts remain below normal values during the first year(s) after allo-HCT (14, 106, 107). This includes abnormally low counts of naive CD4^+^ T cells (persisting for up to two years after allo-HCT), memory CD4^+^ T cells, and CD28^+^CD4^+^ T cells. Conversely, CD8^+^ T cell counts reach the 10^th^ percentile of normal values three to six months after allo-HCT (14, 106, 107). This is due to the faster recovery of memory and CD28^-^CD8^+^ T cells. However, counts of naive CD8^+^ T cells and CD28^+^CD8^+^ T cells remain below normal values up to one year after allo-HCT. Main factors associated with slower T cell reconstitution include graft-versus-host disease (GVHD) and its treatment, administration of anti-thymocyte globulins and/or high dose of MMF for GVHD prophylaxis, and graft-type (slower recovery following cord blood transplantation and faster recovery with peripheral blood stem cell than with bone marrow transplantation). In contrast to T cell recovery, B cell reconstitution after allo-HCT follows their ontogeny in the bone marrow. Very low levels of B cells are detected in the blood in the first two months after allo-HCT (14, 106, 107). Then B cell counts slowly increase to achieve normal values around one year after transplantation. This increase is mainly due to the recovery of naïve B cells. Indeed, memory B cell counts need several years before normalizing. Factors associated with slower B cell recovery include GVHD and its treatment, administration of anti B-cell monoclonal antibodies (such as rituximab, i.e. often given after allo-HCT as treatment for EBV reactivation). In addition, following allo-HCT, levels of blood dendritic cells (and particularly plasmacytoid dendritic cells) are below normal values for up to 6 to 18 months post-transplantation (110). As a consequence of this slow process of immune reconstitution, allo-HCT recipients tend to respond poorly to many vaccines when administered early after transplantation or when they are affected by chronic GVHD (111, 112).

Interestingly, first studies of BNT162b2 vaccine in allo-HCT recipients reported better results than expected, at least in terms of sero-conversion rates (94, 113–115). Specifically, Redjoul et al. assessed anti-spike Ab responses to two doses of the BNT162b2 vaccine given four weeks apart in a cohort of 88 allo-HCT recipients (113). The first dose of the vaccine was administered 3-213 (median 23) months after transplantation. Anti-RBD Ab could be detected in 72 patients (82%) but only 52 had Ab titers > 4160 AU/mL, a threshold defined as a surrogate of protection by the authors based on a prior study showing a good correlation between this threshold and virus neutralization *in vitro* (116). Factors associated with Ab titers > 4160 included absence of immunosuppressive therapy and absolute lymphocyte count > ≥ 1000/μL. Similarly, Ram et al. observed anti-RBD Ab responses to two doses of the BNT162b2 vaccine in 47 of 63 allo-HCT recipients (75%) and in 5 of 14 patients given CD19-based chimeric receptor T cell (CART) therapy (117). Median patient age was 65 years. It should be noted that a T-cell response was demonstrated by ELISpot in three patients with complete B cell aplasia following CART-therapy. More recently, Jimenez et al. found a decreased cellular response to mRNA vaccine in patients under treatment for GVHD (118).

**Figure 2:**
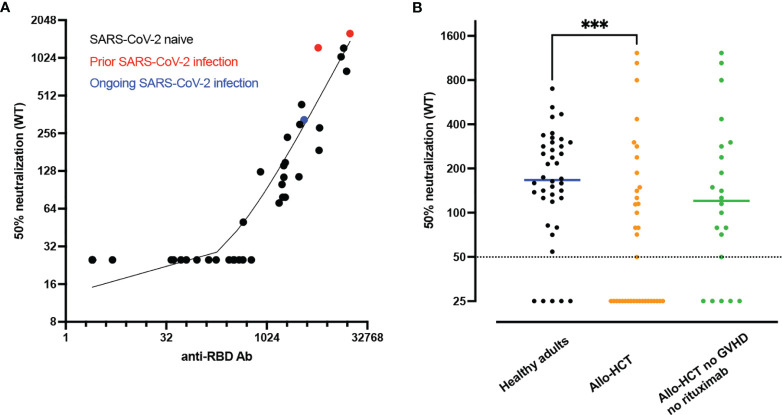
Lower neutralizing antibody (Ab) responses in allogeneic hematopoietic stem cell transplant (allo-HCT) patients than in healthy adults. **(A)** Correlation between anti-RBD Ab and 50% neutralizing Ab titers of SARS-CoV-2 wild type at 28 days after second dose in allo-HCT patients (n=40). The red dots show the data from the two patients with prior SARS-CoV-2 infection while the blue dot shows the data of the patient with ongoing SARS-CoV-2 infection at first vaccination. **(B)** 50% neutralizing Ab titers of SARS-CoV-2 wild type at 28 days after second dose in SARS-CoV-2 naive healthy adults (staff members of nursing homes, n=40, black dots) versus SARS-CoV-2 naive allo-HCT patients (n=37, orange dots) and SARS-CoV-2 naive allo-HCT patients without moderate/severe chronic graft-versus-host disease (GVHD) and rituximab (anti-CD20) administration in the year before vaccination (n=22, green dots). The horizontal solid lines show the median and the broken line shows the lower limit of quantification (LLOQ, 1/50). ***P value < 0.001. Figures from Canti et al. (119).

Assessing neutralizing Ab in immunocompromised patients (including allo-HCT recipients) who are less capable of efficiently producing Ab after vaccination is of particular interest. We investigated baseline clinical and immunological factors predicting anti-RBD Ab titers and neutralizing Ab following two doses of the BNT162b2 vaccine in 40 allo-HCT recipients (median age 60 years) (119). Anti-RBD Ab and neutralizing Ab against wild-type SARS-CoV-2 were detected in 86% and 49% of allo-HCT recipients, respectively. In addition, in comparison to a group of healthy controls, allo-HCT recipients had lower titers of anti-RBD binding Ab as well as lower titers of neutralizing Ab (**Figure 2**). Clinical factors associated with Ab titers and detectable neutralizing Ab included absence of ongoing moderate/severe chronic GVHD and absence of rituximab administration in the 12 months prior to vaccination. Immunological factors associated with neutralizing Ab response included higher counts of memory B cells, of naïve CD4^+^ T cells, and of T_FH_ cells.

### Solid Organ Transplant Recipients

Given that solid organ transplant recipients are at high risk of severe COVID-19 and of death when infected by SARS-CoV-2, a number of studies assessed the response to SARS-CoV-2 mRNA vaccine after solid organ transplantation. Boyarsky et al. reported data from 658 solid organ [kidney (n=322), liver (n=129), heart (n=97), lung (n=71), pancreas (n=5), other multi-organ (n=26)] transplant recipients given two doses of anti- SARS-CoV-2 mRNA vaccine. At four weeks after the second vaccine dose, seroconversion occurred in 357 participants (54%) (120). In univariate analysis there was a lower seroconversion rate in patients <3 years after transplantation (37% versus 67% in those transplanted ≥ 12 years before vaccination, P=0.001), in those under anti-metabolite medications (43% versus 82%, P<0.001), in those patients given the BNT162b2 vaccine (49% versus 60% for the mRNA-1273 vaccine, P<0.001), and in patients > 60 years of age at vaccination (P=0.002).

**Figure 3:**
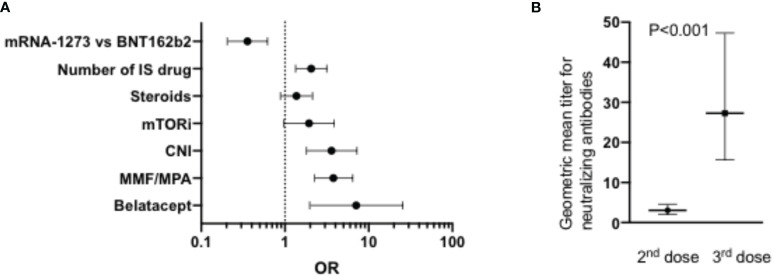
**(A)** Factors associated with failure to achieve detectable Ab after two doses of SARS-CoV-2 mRNA vaccine in renal transplant patients reported by Stumpf et al. (n=368) (121). The graph shows the OR and the Wald CI from logistic regressions. **(B)** Impact of a 3^rd^ dose mRNA vaccine on neutralizing Ab titers in a cohort of 96 heart transplant patients reported by Peled et al. (122). IS, immunosuppressive; mTORi, inhibitors of the mammalian target of rapamycin; CNI, calcineurin inhibitors; MMF, mycophenolate mofetil; MPA, mycophenolic acid.

Another large study compared the response to SARS-CoV-2 mRNA vaccination in renal transplant (n=368, median age 57 years) versus dialysis (n=1256, median age 67 years) patients (121). Seroconversion rates after one and two vaccine doses were 96% and 99% respectively in healthy controls, 62% and 95% respectively in patients on dialysis, and 8% and 42%, respectively, in kidney transplant recipients. Similarly, T-cell response (assessed by IFNγ release assay) was detected in 86% of healthy controls, 78% of patients on dialysis but only 30% of kidney transplant recipients after two doses of the vaccine. In multivariate logistic regression analysis, main factors associated with an absence of seroconversion in dialysis patients were the use of at least one immunosuppressive drug (OR 10; P<0.001), and administration of the BNT162b2 vaccine versus the mRNA1273 vaccine (OR 4.5; P<0.001). Main factors associated with an absence of seroconversion in kidney transplant recipients included older age (P=0.006), shorter time since transplantation (P=0.004), number of immunosuppressive drugs (per one OR 2.0, P=0.001), and administration of the BNT162b2 (versus mRNA1273) vaccine (OR 2.8; P<0.001). The use of the CTLA4 agonist Belatacept was particularly associated with a failure to seroconvert (OR7.1, P=0.003) (**Figure 3**). The negative impact of Belatacept in kidney transplant recipients was confirmed in another large study in which only 1 in 19 (5%) kidney transplant patients on Belatacept had detectable anti-SARS-CoV-2 Ab following two doses of anti-SARS-CoV-2 mRNA (approximately half BNT162b2 and half mRNA1273) vaccine versus 190 on 381 (50%) equivalent non-Belatacept kidney transplant recipients (123). Another study indicated the negative impact of MMF treatment on binding Ab seroconversion and Ab titers, particularly in patients taking at least 1g of MMF per day (124). Specifically, in that study MMF-free regimen was associated with a higher probability of seroconversion (OR=13.25, P<0.001) while there was an inverse correlation between binding Ab titers and MMF concentrations (R=-0.354, P<0.001). A negative impact of MMF on seroconversion was also observed in cohorts of lung and of liver transplant recipients (125, 126). In the latter, high-dose steroids was also associated with a low Ab seroconversion rate (126).

Another large study assessed responses to the BNT162b2 vaccine and to the adenovirus-based ChAdOx1 nCov-19 vaccine (127). In a group of 768 infection-naive patients, the seroconversion rate after two doses of vaccine was 55%. This seroconversion rate was more frequent in patients given the BNT162b2 vaccine (OR=2.5, P<0.0001), in those with tacrolimus monotherapy versus other immunosuppressive regimens (OR=5.2, P<0.0001) and less frequent in patients vaccinated within the year of transplantation (OR=0.28, P=0.0002) and in those with diabetes (OR=0.65, P=0.015). Interestingly, T cell response was identified in 7 out of 40 (18%) infection-naive patients vaccinated with the BNT162b2 and in 2 of 39 patients (5%) given the ChAdOx1 nCov-19 vaccine. As observed also by other groups of investigators (128), seroconversion rates, Ab titers and T-cell responses were significantly higher in COVID-19 recovered compared to naive patients.

A recent study (still in pre-print format) reported an extensive comparison of immunogenicity of anti-SARS-CoV-2 mRNA vaccine in healthy subjects and in 10 kidney transplant recipients (129). The study nicely demonstrated that, in comparison to healthy subjects, kidney transplant recipients had a reduced CD4^+^ T-cell response to the vaccine including both reduced RBD-specific T_H_1 cells and T_FH_ cells. The latter was particularly evident in the draining lymph node. As a consequence, kidney transplant recipients almost failed to mount a SARS-CoV-2-specific germinal center B cell response and to produce RBD-specific memory B-cells (although some spike-specific memory B cells that targeted regions outside RBD were produced) and had a hindered CD8-specific immune response to the vaccine.

Unfortunately, the relatively low response rates to SARS-Cov-2 mRNA vaccine in solid organ transplant recipients has serious clinical consequences. This is illustrated by a recent study that reported 55 cases of breakthrough SARS-CoV-2 infections in fully mRNA vaccinated [BNT162b2 (n=46) and mRNA-1273 (n=9)] solid organ (mainly kidney) transplant recipients (130). These infections occurred a median of 22 (interquartile ranges 13-36) days after the administration of the second vaccine dose. Fifteen the 55 patients required oxygen therapy, six required ICU admission and 3 patients died of COVID-19.

### Patients With Inborn Errors of Immunity (IEI)

Hagin et al. assessed the immunogenicity of the BNT162b2 vaccine in 26 patients with IEI (131). Median patient age was 48 years. Eighteen of them achieved detectable anti-spike Ab after two doses of the vaccine. As expected, the four patients with XLA failed to seroconvert. This was also the case in 2 of 12 patients with common variable immune deficiency (CVID), in 1/1 patient with autoimmune lymphoproliferative syndrome (ALPS)-like disease and in 1/1 patient with combined immunodeficiency. Interestingly, 19 of the 26 IEI patients had detectable T-cell response to S-peptides as measured with an ELISpot assay, including the four patients with XLA. The seven patients without cellular response included 4/12 patients with CVID, two patients with Nuclear factor-kB1 haploinsufficiency and the patient with ALPS-like disease. Amodio et al. confirmed that most patients with CVID or unclassified antibody deficiency achieved a serological response to BNT162.b2 vaccine, although to a lower magnitude than healthy controls (132).

A larger study was reported by Delmonte et al. (133). The study included data from 81 IEI patients (median age 35 years) including six allo-HCT recipients for IEI and 75 patients with ongoing IEI (including 26 patients with STAT3 dominant negative mutations, 14 with autoimmune polyendocrinopathy-candidiasis-ectodermal dystrophy (APECED), seven with CVID, five with hypogammaglobulinemia, and four with warts-hypogammaglobulinemia-infections-myelokathexis (WHIM) syndrome) mostly immunized with mRNA vaccine. Overall response in the 81 IEI patients was good with 85% of the patients having detectable anti-spike Ab after immunization, although their Ab titers were significantly lower than those of healthy controls. Four patients on rituximab for APECED patients (n=3) of for WHIM syndrome (n=1) failed to seroconvert. Interestingly, baseline CD3^+^ T cell count < 1000 cells/µL as well as baseline CD19^+^ cell counts < 100 cells/µL were associated with lower Ab levels post immunization.

More recently, Pham et al. assessed immunization following BNT162b2 or mRNA-1273 vaccine in 33 patients with humoral defects (CVID (n=15), XLA (n=2), hyper-IGM syndrome (n=2), others (n=14) (median age 53 years) (134). While 16/33 patients had detectable RBD-specific binding Ab following vaccination, only two of them had neutralizing Ab. In contrast, the IFNγ release assay was positive in 24 of 31 patients (77%).

## Third Vaccine Doses

It is generally accepted that administering a third vaccine dose is particularly interesting in immunocompromised patients. Indeed, preliminary results suggest that a proportion of patients who failed to achieve seroconversion after two doses of mRNA vaccine do respond to a third dose (122, 135, 136). This is especially relevant given the current omicron variant outbreak (137–139).

Peled et al. investigated the response to a third dose of the BNT162b2 vaccine in a cohort of 96 heart transplant recipients (122). The third dose was administered a median of 168 days after the second dose. Anti-RBD Ab were detected in 23% of the patients after the second dose and in 67% of the patients after the third dose. Further, geometric mean titer for neutralizing Ab increased > 9-fold after the third dose of the vaccine (**Figure 3**). Patients on MMF were less likely to respond to the third dose of the vaccine.

Benotmane et al. investigated the impact of a third-dose of the mRNA-1273 vaccine in kidney transplant recipients without seroconversion (n=95) or with low (arbitrarily defined as < 50 AU/mL) anti-spike IgG titers (n=64) after two doses of the vaccine (135). The third dose was given 48 to 59 days after the second one. Seventy-eight patients (49%) had anti-spike IgG titers > 50 AU/mL 28 days after the third dose including 27% of patients without detectable response after two doses of the vaccine. In another French cohort of kidney transplant recipients, a third-dose of BNT162b2 vaccine induced a seroconversion in 34 out of 85 patients (40%) who were seronegative after two doses of the vaccine (136). Unfortunately, administration of a third dose of the vaccine was not efficient in patients on belatacept therapy (140). Importantly, the third vaccine dose increased not only Ab titers but also spike-reactive B and CD4^+^ T cells (141).

These observations were confirmed in a recent randomized double-blind study comparing third dose mRNA-173 vaccine versus placebo in a cohort of 120 solid-organ transplant patients (142). A third vaccine dose or a placebo was administered a median of two months after the second dose of the vaccine and no patients had prior COVID-19. Ab levels > 100 U/mL were observed in 55% of the patients in the mRNA-173 group versus 18% in the placebo group (P<0.001). Further, patients randomized in the mRNA-173 group were more likely to have detectable neutralizing Ab (60% versus 25%; relative risk= 2.4; 95% CI, 1.5 - 4.0) and had significantly higher frequencies of spike-specific CD4^+^ T cells.

However, importantly, two recent studies demonstrated that 25-68% of solid organ transplant recipients had detectable neutralizing Ab against the Delta variant after the third dose of mRNA vaccine (versus 100% of healthy controls) (143, 144). Further, none of 51 kidney transplant recipients had detectable neutralizing Ab against the Omicron variant (144).

Administration of a third dose of the BNT162b2 vaccine was also studied in allo-HCT recipients. In a first cohort of 42 allo-HCT recipients with “low” anti-RBD IgG titers [defined as < 4160 AU/mL, as discussed above a threshold proposed as protective by the authors (113)], the third vaccine dose was administered 51 ± 22 days after the second dose (145). Although the mean Ab titer increased from 737 ± 1009 to 11099 ± 18607 AU/mL, only 20 of the 42 patients achieved Ab titers > 4160 AU/mL. Patients with higher B cell count and those with Ab titers > 1000 AU/mL at the time of the third vaccination were more likely to achieve Ab titers > 4160 AU/mL after the third dose. More recently, Maillard et al. investigated the impact of a third vaccine dose in 181 allo-HCT recipients (146). The third vaccine was given a median of 54 days after dose two. Twenty-nine of 70 patients (41%) without Ab response after the first two doses seroconverted while the booster vaccine significantly increased binding Ab titers in remaining patients. Unfortunately, neutralizing Ab against variants of concern were not assessed in this study.

Third dose vaccination has also been investigated in patients suffering from a hematological malignancy. Herishanu et al. investigated the impact of a third-dose BNT162b2 administration in a cohort of 172 patients with chronic lymphocytic leukemia who had no serological response after two doses of the vaccine (147). Median patient age was 72 years and the median time between second and third vaccination was 179 days. Anti-spike binding IgG response to the third dose was seen in only 41 of 172 patients (24%). Factors predicting the absence of seroconversion after the third dose included active therapy, anti-CD20 therapy in the year before the third vaccine administration, age > 65 years, and IgA levels < 80 mg/dL. Finally, Terpos et al. investigated the impact of a third dose of BNT162b2 in a cohort of 167 patients with multiple myeloma (median age 68 years) (148). Among 57 patients with neutralizing Ab titers < 30% at third dose (measured with a surrogate virus neutralization test), 32 patients (56%) patients achieved neutralizing Ab titers ≥30% including 26 patients (45.6%) achieving neutralizing Ab titers of ≥50% after the booster dose. Patients on anti-BCMA therapy had a much lower probability of achieving detectable neutralizing Ab (OR=0.03, 95% CI: 0.003-0.27).

## Future Directions

### Temporary Hold of Immunosuppressive Medications

As reviewed above, several studies observed that some immunosuppressive drugs (such as MMF or CTLA4 agonists) are associated with impaired responses to mRNA vaccines. This prompted the American College of Rheumatology to propose guidelines on how to manage the use of immunosuppressive medication, whenever feasible for the underlying disease, to improve the response to SARS-CoV-2 mRNA vaccines (149). These guidelines proposed to temporarily hold MMF, methotrexate or JAK inhibitors for one week following each vaccine dose. For abatacept administered s.c., the recommendations consisted of holding the drug one week prior to and one week after the first vaccine dose only. For abatacept administered i.v., the guidelines proposed to administer the first dose of the vaccine four weeks after abatacept infusion and to administer the next dose one week after vaccination (gap of five weeks between the two abatacept doses).

Picchianti-Diamanti et al. assessed the efficacy of the BNT162b2 vaccine in a cohort of 35 patients with rheumatoid arthritis (150). Ongoing rheumatoid arthritis medications were adapted during the vaccination period according to the guidelines described just above. Importantly, no rheumatoid arthritis flare was observed. Following vaccination, anti-RBD Abs were detected in 34 of the 35 patients while spike-specific T cell responses were detected in 24 of the 35 (69%) patients versus in all controls. These early data suggest that holding immunosuppressive drugs during vaccination as recommended above might improve its efficacy.

### Fourth Dose Vaccination

A relevant question is whether patients who failed to respond to three doses of the vaccine can respond to a fourth one. This was studied by Kamar et al. in a cohort of 37 solid organ transplant recipients, including five patients who had a weak response to the previous three doses (Ab <14 BAU/mL and 31 patients who had no response to the first three doses (151). Thirteen of 31 seronegative patients seroconverted with the fourth dose while median Ab concentration increased from 4 BAU/mL to 402 BAU/mL in the five remaining patients.

In a preprint format, Schrezenmeier et al. reported the result of a study assessing a fourth dose BNT162b2 vaccine in 29 kidney transplant recipients who did not seroconvert with the three first doses of the vaccine and were on MMF (152). The fourth dose was administered 4-7 days after MMF hold and MMF was paused until day 28-35 after vaccination. Remarkably, 76% of the patients seroconverted with this strategy. Importantly, no signs of organ rejection were observed.

## Summary

**Figure 4:**
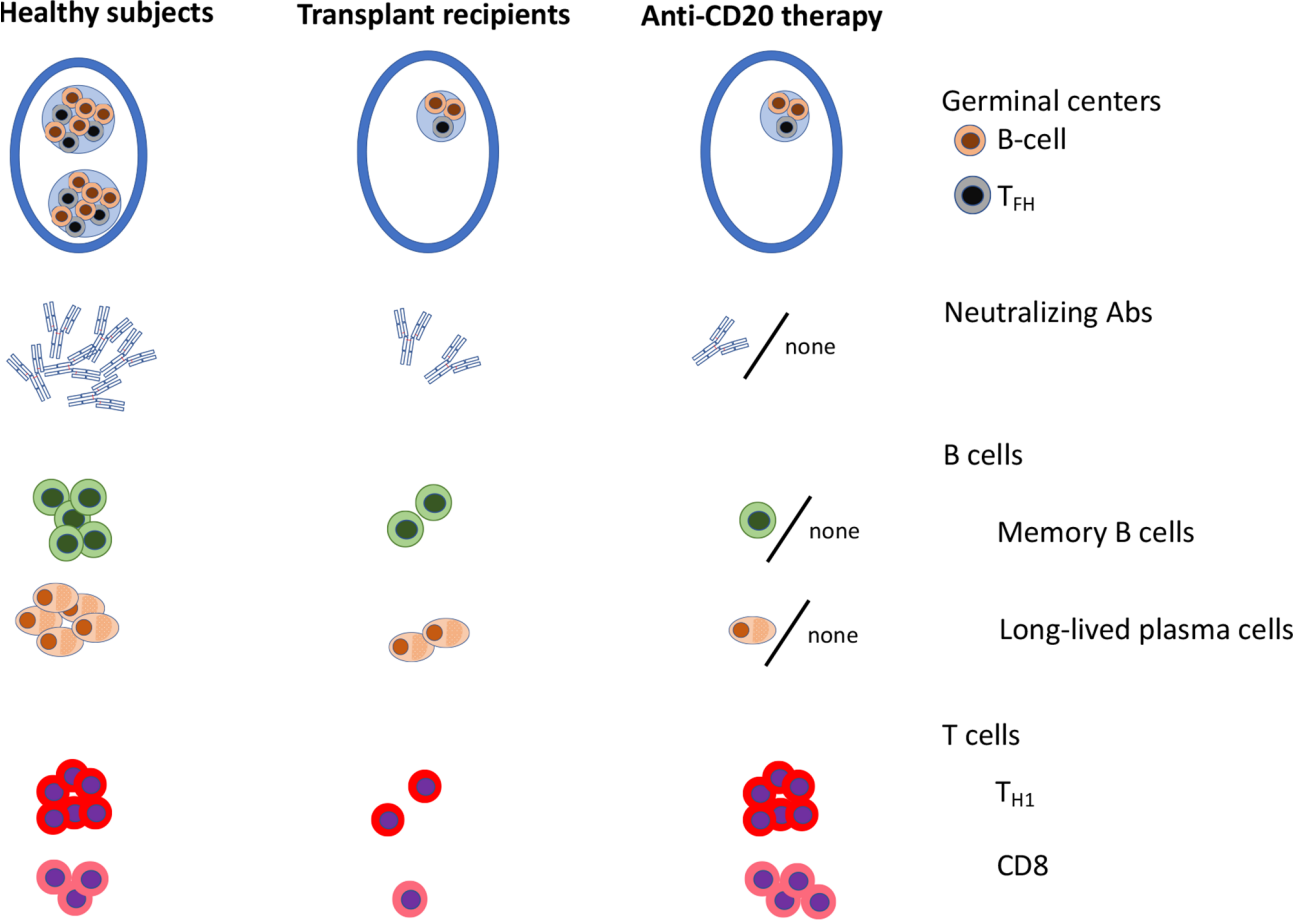
Graphical representation of anti-SARS-CoV-2 mRNA vaccine immunogenicity in healthy subjects, in solid organ transplant recipients and in patients on anti-CD20 therapy (93, 129). TFH, follicular helper T cells; Th1, type 1 helper CD4+ T cells.

Early clinical mRNA vaccination trials in immunocompromised patients have demonstrated lower seroconversion rates and lower Ab titers in immunocompromised patients as compared to age-matched healthy subjects. Yet, many immunocompromised patients are successfully immunized with two doses of mRNA vaccine. Common predictors of poor Ab response have been identified (**Table 1**). These include agents blocking co-stimulation signals, agents or conditions associated with B cell defects (anti-CD20 therapies, CD19-targeting CART-cells, anti-CD38 therapy, Bruton’s tyrosine kinase inhibitors or XLA), conditions associated with deficit of naive CD4^+^ T cells (such as allo-HCT recipients), and agents targeting lymphocyte proliferation or function such as antimetabolites and to a lesser extent JAK inhibitors, calcineurin inhibitors or steroids. In patients with poor Ab responses to the mRNA vaccine because of pure B cell defects, a higher cellular CD8^+^ T cell response has been observed (**Figure 4**). Whether this is sufficient to protect the patients from severe COVID-19 remains to be demonstrated. In contrast, after allo-HCT or solid organ transplantation, some patients experience both poor Ab and cellular response, a condition which is similarly encountered after antimetabolite or CTLA4 agonist therapy (**Figure 4**). Interestingly, several studies observed better seroconversion rates with the mRNA1273 than with the BNT162b2 vaccine while some studies showed better immunogenicity of the BNT162b2 in comparison to the ChAdOx1 nCov-19 vaccine. Importantly, early data suggest that the administration of a third/fourth vaccine dose might help patients with detectable but low Ab titers following two doses of the vaccine. Whether the level of protection achieved is sufficient to prevent infection with the omicron variant is currently the focus of several ongoing investigations. Further, early results observed with a temporary hold of immunosuppressive drugs are also encouraging. As an alternative to vaccination, a phase III study is currently investigating the prophylactic administration of anti-spike mAb in immunocompromised patients who do not respond to SARS-CoV-2 vaccines (clinicaltrials.gov NCT05074433). The limitation of this last approach, however, is that the Omicron variant partially or totally escapes neutralization by many of currently available mAbs (153). Finally, further investigation using for instance a systems vaccinology approach might be useful to identify pathways preventing seroconversion in immunocompromised patients and further improve mRNA vaccine efficacy in vulnerable populations.

**Table 1:** Factors predicting failure to achieve anti-spike binding Ab following two-doses of mRNA vaccine in selected studies of SARS-CoV-2 mRNA vaccination in immunocompromised patients.

Condition	% of patients who failed to seroconvert	Factors associated with failure to seroconvert
People living with HIV (15, 82, 84)	2-12	- CD4+ T-cell counts < 200 cells/μL (15, 84) - BNT162b2 versus mRNA-1273 vaccine (84)
Chronic inflammatory disease (85, 86, 89, 91)	11-18	- Age > 65 years (85) - anti-CD20 (85, 86, 89, 91) - glucocorticoids (85, 86, 89) - CTLA4 agonist (85, 89) - MMF (85, 89) - Methotrexate (91) - JAK inhibitor (89)
Chronic lymphocytic leukemia (98–100)	33-60	- Age > 65 years (98, 100) - Male (98) - Active CLL treatment (particularly BTKi and anti-CD20 within 1 year of vaccination) (98–100) - Low gammaglobulin or low IgG levels (98, 100) - BNT162b2 versus mRNA-1273 vaccine (100)
B-cell non-Hodgkin lymphoma (101, 102)	51-58	- Time since last anti-CD20 < 9 months (101, 102) - ALC < 900 cells/μL (101, 102)
Myeloproliferative neoplasm (154)	14	- diagnosis of myelofibrosis (even untreated) - JAK inhibitor
Multiple myeloma (103, 104)	16-22	- Absence of complete remission - BCMA-targeted treatment - anti-CD38 monoclonal Ab
Allogeneic hematopoietic stem cell transplantation (113, 115, 117, 119, 146, 155, 156)	14-25	- ALC < 1000 cells/μL (113, 146) - Systemic immunossupressive treatment in the last 3 months (113, 115, 146) - anti-CD20 therapy in the year before vaccination (119, 146) - Time from transplantation to vaccination < 1 year (146) - moderate/severe chronic GVHD (115, 119, 146) - low memory B cell counts (119) - low naive T cell counts (119)
Solid organ transplantation (120, 121, 123–125, 127)	46-82	- Short time between transplantation and vaccination (120, 121) - Number of immunosuppressive drugs (121) - Anti-metabolite medication (120, 124) - mTOR inhibitor (125) - CTLA4 agonist (121, 123) - BNT162.b2 versus mRNA-1273 vaccine (120, 121) - ChAdOx1 versus BNT162b2 vaccine (127) - Older age (120, 121) - CD4 counts < 400 cells/μL (157) - Lung transplantation (125)
Inborn errors of immunity (131, 133, 134, 155)	15-51	- XLA (no response as expected) (131, 134, 155) - APECED (133) - anti-CD20 therapy (133) - CD3 counts < 1000 cells/μL (133) - CD19 counts < 100 cells/μL (133)

## Author Contributions

FB is first author and AM and SH-B are co-senior authors. All authors contributed to the article and approved the submitted version.

## Funding

FB is Senior Research Associate, LC a Télévie Research fellow, and AM Research Director at the National Fund for Scientific Research (FNRS) Belgium. This manuscript has been supported by the following grants: FB: Crédits Sectoriels de Recherche en Sciences de la Santé (FSR 2021) from the University of Liège; FB Grant T.0016.20 from the FNRS; KA: Flemish Research Foundation grant number FWO G0G4220N; MG is supported by a fellowship from the Belgian kid’s fund; SH-B: European Union’s Horizon 2020 research and innovation program (grant agreement Nos. 874707 “EXIMIOUS”), PRISMA project funded by VLAIO (Flanders Innovation & Entrepreneurship), and Stichting Alzheimer Onderzoek -Fondation Recherche Maladie Alzheimer (SAO-FMA) and KU Leuven starting grant.

## Conflict of Interest

FB has received travel grants and/or speaker honoraria from Celgene, Abbvie, Novartis, Pfizer and Sanofi.

The remaining authors declare that the research was conducted in the absence of any commercial or financial relationships that could be construed as a potential conflict of interest.

## Publisher’s Note

All claims expressed in this article are solely those of the authors and do not necessarily represent those of their affiliated organizations, or those of the publisher, the editors and the reviewers. Any product that may be evaluated in this article, or claim that may be made by its manufacturer, is not guaranteed or endorsed by the publisher.
